# Review of the Neural Processes of Working Memory Training: Controlling the Impulse to Throw the Baby Out With the Bathwater

**DOI:** 10.3389/fpsyt.2020.512761

**Published:** 2020-09-10

**Authors:** Samantha J. Brooks, Rhiannon Mackenzie-Phelan, Jamie Tully, Helgi B. Schiöth

**Affiliations:** ^1^ School of Psychology, Faculty of Health, Liverpool John Moores University, Liverpool, United Kingdom; ^2^ Section of Functional Pharmacology, Department of Neuroscience, Uppsala University, Uppsala, Sweden; ^3^ Neuroscience Research Laboratory (NeuRL), Department of Psychology, School of Human and Community Development, University of the Witwatersrand, Johannesburg, South Africa; ^4^ Institute for Translational Medicine and Biotechnology, Sechenov First Moscow State Medical University, Moscow, Russia

**Keywords:** working memory training, neuroplasticity, n-back, CogMed, frontoparietal, striatal, addiction

## Abstract

**Background:**

Smartphone technology has enabled the creation of many working memory training (WMT) Apps, with those peer-reviewed described in a recent review. WMT claims to improve working memory, attention deficits, hyperactivity and fluid intelligence, in line with plasticity brain changes. Critics argue that WMT is unable to achieve “far-transfer”—the attainment of benefits to cognition from one taught context to another dissimilar context—associated with improved quality of life. However, brain changes after a course of WMT in frontoparietal and striatal circuits—that often occur prior to behavioral changes—may be a better indicator of far-transfer efficacy, especially to improve impulse control commonly dysregulated in those with addictive disorders, yet not commonly examined in WMT studies.

**Method:**

In contrast to previous reviews, the aim here is to focus on the findings of brain imaging WMT training studies across various imaging modalities that use various paradigms, published *via* PubMed, Scopus, Medline, and Google Scholar.

**Results:**

35 brain imaging studies utilized fMRI, structural imaging (MRI, DTI), functional connectivity, EEG, transcranial direct current stimulation (tDCS), cerebral perfusion, and neurogenetic analyses with tasks based on visuospatial and auditory working memory, dual and standard n-back.

**Discussion:**

Evidence suggests that repeated WMT reduces brain activation in frontoparietal and striatal networks reflective of increased neural circuitry efficiency *via* myelination and functional connectivity changes. Neural effects of WMT may persist months after training has ended, lead to non-trained task transfer, be strengthened by auxiliary methods such as tDCS and be related to COMT polymorphisms. WMT could be utilized as an effective, non-invasive intervention for working memory deficits to treat impulse and affective control problems in people with addictive disorders.

## Introduction

Working memory training (WMT) is a “do-no-harm” cognitive alternative (with less side effects, and greater home-based accessibility) to existing psychotherapy and pharmacotherapy for various impulse control deficits [e.g., attention deficit hyperactivity disorder (ADHD), substance use disorder, behavioral addictions and eating disorders] (1). It is proposed that WMT harnesses inherent neuroplasticity mechanisms within frontoparietal and striatal circuits, to evoke better self-regulation—via the holding in mind of cognitive strategies—over a training period of approximately 1 to 2 months (2). Extensive reviews to date examining the potential mechanisms and outcomes of WMT have concluded that most WMT paradigms are effective at “near transfer”—the ability to evoke improved performance on the trained or related task (3). However, evidence of “far-transfer”—the ability of WMT to improve cognitions and behaviors on non-trained tasks that enhance quality of life—have been less convincing, leading to the speculation that training improvements on specific WMT do not alter neural processes in a clinically relevant, long-term manner. Indeed, far-transfer of WMT to broad quality of life factors are difficult to identify, given that publications have focused predominantly on measures of working memory performance (accuracy, response times) related to attention in children with ADHD (4–7) or with Autism Spectrum Disorder (8); in adults (3, 9–14); particularly older adults (15–22). Additionally, some studies have examined the effects of WMT on dysphoria (23), cognition in children with Fragile X syndrome (24), cognition in adults with HIV (25), and football players aiming to improve their performance (26). Furthermore, the reliability and validity of brain imaging measures to quantify far-transfer effects of WMT must be examined cautiously (e.g., with advanced neuroimaging techniques, such a functional connectivity and multiple regression analyses), given the myriad of individual differences and indirect influences on vasculature function that contribute to measures of neural structure and function.

Consequently, this begs the question as to whether WMT is indeed a redundant paradigm (27) or whether it should be at least substantially revised with more advanced imaging techniques, or incorporated into other related training paradigms, such as individualized cognitive remediation therapy (28). On the other hand, one could consider whether exclusive reliance on primary outcomes associated with working memory and attention measures is shrouding the far-transfer or longevity effects of WMT, and whether the time is now ripe (before “*throwing the baby out with the bathwater*”) to consider other potentially effective measures. Furthermore, perhaps, the currently accepted definition of a far-transfer effect is too narrow, in that it does not consider how distant aspects of cognitive performance and behaviors (i.e., working memory performance) might enhance seemingly unrelated functions (i.e., impulse control) which, in turn, impact quality of life (29). WMT paradigms might therefore have potential far-transfer benefits which have not been previously considered, and which might best be understood by examining the extant neuroimaging literature.

With the technology and sophisticated software that non-invasive brain imaging methods offer, uncovering neural effects is all the more feasible in populations that would benefit from non-invasive, non-pharmacological brain-focused interventions, such as those with impulse control disorders (e.g., addictions), where interventions are currently ineffective for relapse rates. As such, marked changes in activity across areas of the brain associated with specific behavior changes could infer a positive treatment outcome and far-transfer effect. For example, those with addictive disorders who often demonstrate profound deficits in impulse control, show dysfunctional frontostriatal functioning (30) with improvements in impulsivity and working memory observed within similar neural circuitry (31). That said, one could also argue that impulse control and improvements following WMT share few neural substrates, which would highlight that far-tranfer associated with impulse control is difficult to show with neuroimaging data. For example, according to the neurosynth brain imaging meta-analysis tool (neurosynth.org) a search conducted in August 2020, using the term “working memory”, yielded a meta-analysis of 1,091 studies, demonstrating significant activation predominantly in the bilateral dorsolateral prefrontal cortex (DLPFC), bilateral insula, and anterior cingulate cortex. Conversely, a search using the term “impulsivity” included 120 studies and demonstrated less significant regional activation in the dorsal striatum only. Thus, it appears that working memory function is predominantly a “top-down” function associated with prefrontal cortex activation, whereas impulsivity is predominantly a “bottom-up” function associated with striatal activation. Nevertheless, despite the dissociation between these two functions, it has well been established that efficient working memory processing is associated with effective frontostriatal activity and reduced impulsivity (32–34).

According to our recent review (1) CogMed (35) WMT has the highest number of publications to date, aiming to improve the neural mechanisms underlying attention deficits in daily life and in those with ADHD (36). CogMed is a computerized, multitask, clinician-led training intervention adapted for both children and adults, as well as healthy and disordered populations—which may be a “possibly efficacious treatment” for ADHD symptoms in youth (5), but perhaps only in combination with pharmacotherapy, which is problematic for some (6). However, in a recent meta-analysis of over 750 children with ADHD engaging in cognitive training, un-blinded raters most proximal to the training session were most likely to report significant outcomes than blinded raters, therefore suggesting an experimenter bias (37). Moreover, whereas the effects on hyperactivity, impulsivity, and academic performance were not significant in the meta-analysis of Cortese et al., the near-transfer effects on working memory and related executive functions were consistent with WMT findings in other domains (and other populations not included in their meta-analysis).

Similar reviews have criticized CogMed for not sufficiently substantiating claims that WMT improves attention and ADHD symptoms (38). Furthermore, in recent reviews of the efficacy of all types of cognitive training listed on the “cognitivetraining.org” website, there is extensive evidence that brain-training interventions improve performance on trained tasks, less evidence of improvements on closely related tasks, and little evidence that training enhances performance on distantly related tasks, or that training improves everyday cognitive performance (39). That said, there is scant evidence of specific impulse control improvements following WMT, a cognitive skill closely related to working memory and frontostriatal circuitry that is typically dysregulated in those with addictions and appetite control disorders (33).

Thus, from the perspective of current reviews and meta-analyses of WMT, it appears that the field runs the risk of becoming at worst redundant and unable to make a sustained impact on the neuroscience of mental health and enhancement of cognitive ability (27). However, other reviews—particularly of neuroimaging data—demonstrate another perspective, such as a recent paper on the effects of CogMed on neural processes (2). CogMed for those with attention deficits appears to evoke significant structural and functional changes within working memory networks and related regions, such as decreased activity in frontoparietal networks and greater connectivity between the prefrontal and parietal cortex (2). There is, however, some debate as to the effect that WMT has on brain functioning, with acute repetition training (e.g., in one session) evoking increased activation, whereas longer training durations over weeks are related to decreased activation (40–43), suggestive of priming or practice effects versus neuroplasticity effects. Moreover, there are well-known working memory capacity reductions with age, associated with reduced striatal functioning that subserve effective updating of information received (43), yet it is still not yet clear how WMT differentially effects brain functioning in younger versus older people. In addition, frontoparietal and frontostriatal circuits that are altered or made more efficient by WMT are also often dysfunctional in populations experiencing behavioral addictions (e.g., pathological gambling, excessive internet use, and eating disorders) and substance use disorders (e.g., alcohol use disorder, and stimulant misuse) (33, 44, 45), and so WMT would seem a useful non-invasive intervention to improve these neural processes. Furthermore, various psychiatric populations undergoing treatment or at risk of developing a psychiatric disorder demonstrate neurocognitive changes prior to significant behavioral or physical changes, which would support the notion that WMT may alter neurocognitive processes before behavioral far-transfer becomes apparent. In this vein, a recent WMT fMRI study of ADHD suggests that brain changes occur before significant behavioral changes (46).

Against this background, with the view that there may still be validity in pursuing WMT for treating the neural processes underlying impulse control deficits, three points are considered in this review:

- Alternative approaches to measure the effectiveness of WMT across various training paradigms include brain imaging measures—such as structural and functional MRI—which appear to demonstrate neural changes often independently of behavioral changes that may occur later.
- WMT research has so far relied on measures of ADHD symptoms, working memory performance (e.g., accuracy and response time), and global intelligence/executive function improvements, which critics argue do not elicit significant far transfer effects (27, 47, 48). However, exploration with various brain imaging modalities to measure WMT efficacy, particularly in relation to the neural circuitry underlying impulse control (32, 33, 49) may yield significant findings for those with addictive disorders.
- Measures of the neural correlates of impulse control in relation to WMT in those with addictive disorders (32, 33, 37, 49) are currently scarce.

With these points in mind, the first aim of the current review is to examine all brain imaging findings across all peer-reviewed WMT paradigms since the first neuroimaging WMT study (40) to the present. The second aim is to explore the claim that there is a paucity of measurement of impulse control (associated with working memory deficits in those with addictive disorders) in neuroimaging studies of WMT. Given that near and far transfer effects on working memory, ADHD symptoms, attention, and global intelligence have been extensively reviewed elsewhere (27, 50) and that the last neuroimaging WMT review focused on CogMed (2), this review will focus on brain imaging studies across all paradigms and neuroimaging modalities. Of note, the most recent meta-analysis of WMT (not a review of neuroimaging studies) concludes that small significant and long-lasting gains occurred for working memory, which are moderated by the type of training tasks, the adopted outcome measures, the training duration, and the total number of training hours (50). Describing the outcomes of individual WMT paradigms is outside the scope of this review but can be found in various recent reviews (1, 27, 50). However, it is pertinent to note that the founder of CogMed (51) concludes that a total amount of at least 8 hours of WMT or a training period of at least 3 weeks is needed to produce significant training effects in the brain. Against this background, there is as yet no review of all WMT paradigms that have been used in neuroimaging studies of various modalities, with various populations, and so the current review addresses this gap in the literature.

## Methods

**Figure 1:**
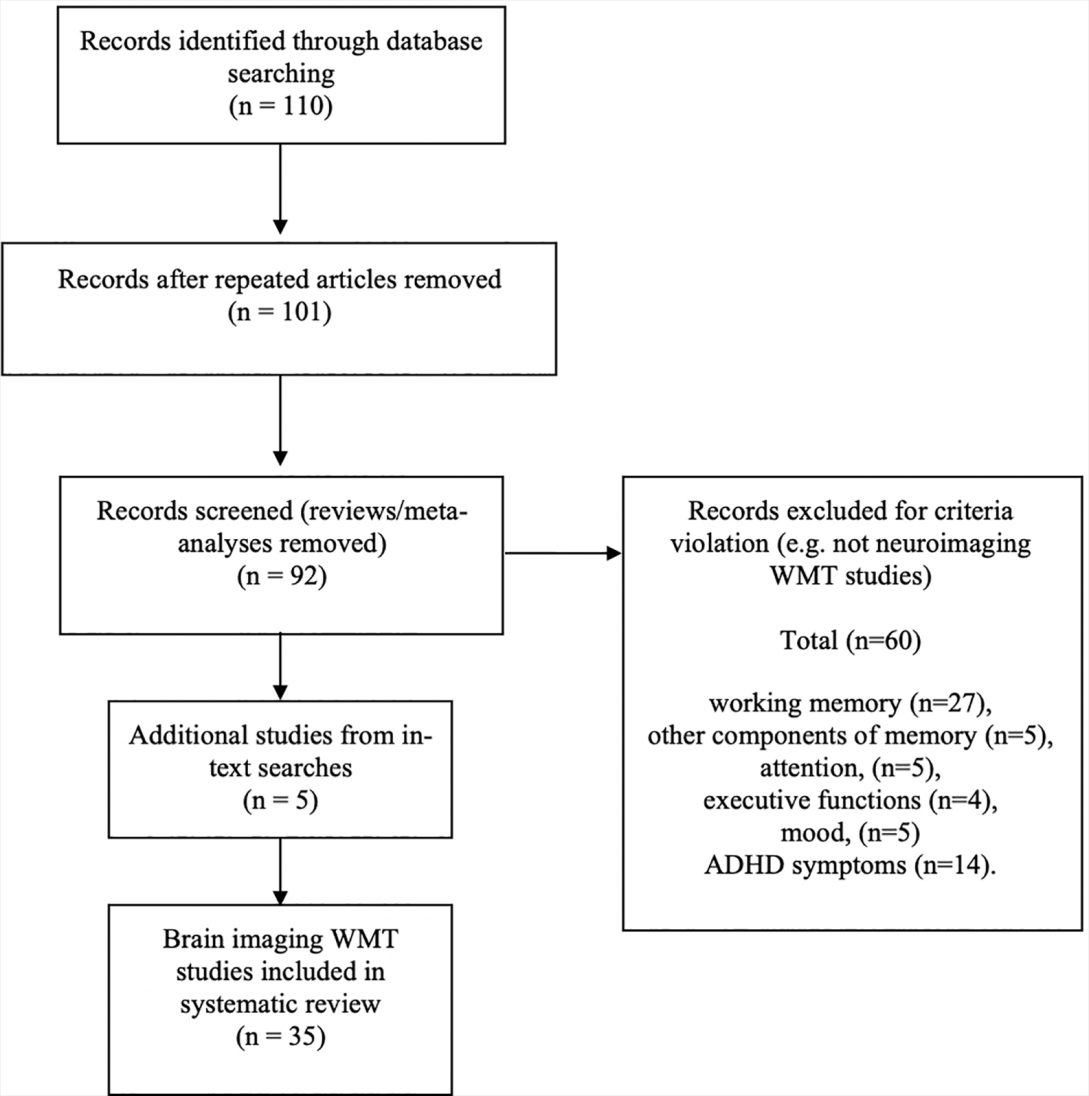
CONSORT diagram.

See **Figure 1** for a CONSORT diagram describing the search criteria. The following online platforms were searched for relevant articles: Google, Google Scholar, Pubmed, Medline, and ScienceDirect, with a search using the phrase “working memory training” + the name of each of the WMT paradigms included in the previous review (1). Inclusion criteria for neuroimaging studies were: a) any neuroimaging modality article written in English; b) WMT programmes supported by published peer review focusing on brain imaging methods; c) original articles and not reviews or meta-analyses (although these are referred to in text); d) publications that report the outcomes of training and not a protocol for a future WMT study; e) articles published since the first WMT brain imaging study (40) up to September 2019; f) only WMT and no other forms of cognitive training were included. Additionally, references from reviewed papers were examined.

## Results

Thirty-five neuroimaging WMT studies were found using task-based functional magnetic resonance imaging (task-based fMRI: n = 20, including 3 studies examining COMT), resting state fMRI (n = 2); structural imaging [magnetic resonance imaging (MRI), diffusion tensor imaging (DTI), and connectome voxel-based fractional ansiotrophy, n = 3], transcranial direct current stimulation (tDCS, n = 6), electroencephalography (EEG), cerebral perfusion (n = 4). WMT neuroimaging studies found in this review utilized visuospatial and auditory working memory tasks, dual or standard n-back tasks (some of which formed interventions, such as CogMed™, C-Ya™, and ACTIVATE™).

### Functional fMRI

This technique is an imaging modality which measures neuronal activation by detecting changes in blood flow throughout the brain in a process termed neurovascular coupling, using a measure known as *blood oxygen level dependency (BOLD)*. When an area of the brain is active, haemodynamic activity in the region increases, enabling fMRI to measure changes in neuronal activation (52). Research with WMT has primarily relied on task-based brain activation methods, but some studies also use resting state functional connectivity methodologies which are described below.

#### Task-Based fMRI Studies

The majority of neuroimaging studies of WMT to date have utilized fMRI measures, currently including 18 fMRI studies. The first brain imaging study of WMT used fMRI and was conducted two decades ago by Garavan and colleagues in 2000 (40). Using a moderate or extensive short-repetition training intervention with the visuospatial delayed match-to-sample working memory task, the effects of WMT on functional neuroanatomy were examined. Decreased activation after a short period of training was reported in frontoparietal, cingulate, insular, and occipital cortex, which the authors, at this early stage of WMT research, suggested was an indication of practice effects and not neural reorganization. However, in line with two decades of subsequent neuroimaging research with various modalities, the Garavan et al. study may rather suggest improvements in neural efficiency. Landau and colleagues also observed neural deactivation after WMT in an event-related fMRI task and suggest that reduced activation in frontoparietal regions reflects greater encoding efficiency over the time course of training (42).

A potential inverted-U function was proposed to further explain the activation versus deactivation debate in WMT neuroimaging studies. This was done by another early fMRI study of WMT where the authors suggest that timing differences may occur in cerebral reorganization after WMT (41). In their study, Hempel and colleagues show increased activation in the right inferior frontal and right intraparietal cortices after two weeks of standard, increased load n-back training, but reduced activation during consolidation and after a month. Another early WMT fMRI study to examine timing effects was conducted by Olesen and colleagues (53), whereby healthy adults practiced a variety of working memory tasks for 5 weeks (tasks that would become the CogMed package), and their brain activations before, during and after training were measured. The authors reported that after training, activation increases associated with working memory were observed in the middle frontal gyrus and the inferior/superior parietal cortices. The authors argue that, whereas other studies show decreased activation following training, which may be a result of automation of cognition, practice effects, reduced cognitive load and consolidation effects over time, increased activation may rather be reflective of independence from such priming effects and new connectivity between brain regions.

However, similarly to Garavan’s study (as well as later studies), Salaya and colleagues in 2006 (54) argue that in contrast to training resulting in an automatic stimulus-response association or better encoding, WMT instead relies on a continuous cognitive control process during repeated performance that leads to greater neural efficiency. In line with this suggestion, Salaya and colleagues found decreases in neural activation in both spatial and object-selective brain areas (e.g., frontal, parietal, and insular cortices) after spatial WM task repetition (and not the object-recognition task) that was independent of behavioral performance. The authors’ concluded that repetition of spatial WMT in particular enabled greater neural efficiency in processing task-relevant information and improved filtering of task irrelevant stimuli. At the same time, Klingberg and colleague conducted single subject analyses using fMRI study of three subjects, to closely examine cortical reorganization over time after training with CogMed (55). The authors found that increased activation associated with WMT could be attributed to a wider recruitment of frontoparietal cortex regions during the course of training (maximum 5 weeks). Adding to the debate concerning increased or decreased activation and relative training gains, Dahlin and colleagues found, with a variable load n-back task that relative striatal activation differences between young versus older participants contributed to transfer of cognitive gain to an unrelated task (43). In other words, after 5 weeks of WMT, the study found that letter recall ability (a task unrelated to the WMT) was dependent on the degree of pre-training striatal activation, and also that older-age decline in striatal activation reduced transfer gains.

In another early fMRI study of WMT by Schneiders and colleagues in 2011 (56) the neural correlates of visual versus auditory training was examined. Greater training gains were reported with visual—as opposed to auditory—N-back (2-back), specifically in decreased activation in the right middle frontal gyrus and right posterior parietal lobe. The authors concluded that the findings suggest intra-modal but not cross-modal neural efficiency after training. Schneider and colleagues conducted another fMRI study a year later to examine whether similar effects could be observed in auditory training only, using a tonal n-back task (57). Decreased activation in the right inferior frontal gyrus and right inferior parietal lobe was again found, suggesting a cross-modal effect and reduced demand on general attentional processes after WMT, independent of behavioral performance. In a study by Schweizer and colleagues (58) participants engaged in an affective daily adaptive dual n-back task, for approximately 20 sessions between 20 and 30 min. During the task, emotionally neutral or emotionally negative faces and words were shown. Before and after the intervention, participants were scanned on the trained task at 1 back, 2 back, 3 back, and 5 back levels. During the 3-back condition, activation decreases were observed in the left dorsolateral prefrontal cortex, right superior frontal gyrus, left, and right supramarginal gyrus, left and right middle temporal gyrus, and left and right middle occipital lobe. Conversely, during the more difficult 5-back condition, activation increases were found in the right orbitofrontal cortex, right inferior frontal gyrus, and right inferior parietal cortex, suggesting neural efficiency gains at the lower cognitive load, combined with greater neural activity needed to cope with greater demands (perhaps, until the higher cognitive demands are mastered). Heinzel and colleagues in 2014 conducted a WMT fMRI study using various n-back loads to examine the sigmoid or inverted U pattern of neural activation as working memory load increases, in young versus older people (59). It has been suggested that accelerated changes in this neural pattern occur as a function of age-related changes in working memory capacity and neural efficiency. Heinzel and colleagues examined this and found increased neural efficiency and capacity after WMT, which they termed more “youth-like” brain response patterns in frontoparietal circuitry. In turn, this activation was associated with better training scores independent of the effects of age, gender, education, gray matter volume, and baseline working memory performance. In addition, reduced activation was found after training on lower cognitive loads. The authors conclude that cognitive benefits in neural activation were demonstrated in older adults who engaged in WMT with increasing loads.

Another fMRI study by the same group examined the effect that WMT has on brain activation in relation to maintaining and improving cognition in older age (60). The authors continued their investigations as to whether frontoparietal activations in older participants (between age 60 and 75 years) can be improved with WMT and related to transfer of gains to non-trained cognitive tasks. To do this, Heinzel and colleagues examined neural correlates associated with WMT transfer benefits on the untrained Sternberg delayed recognition task. It was found that WMT transfer benefits on the Sternberg task in older adults was related to decreased activation in the right lateral middle frontal gyrus/caudal superior frontal sulcus. Heinzel and colleagues conclude that their fMRI findings indicate greater processing efficiency in neural circuits supporting working memory updating ability that was able to be transferred to a delayed recall task in older adults.

Progressing this work more recently, Heinzel and colleagues examined transfer effects to a multimodal dual task, to examine whether working memory can be improved in older adults (60–72 years of age), and whether transfer gains occur across visual and auditory modalities (20). In their study, adults simultaneously completed delayed match to sample auditory or visual working memory tasks that were performed either in isolation (single-task) or in conjunction (dual task). It was found that neural activity changes in left DLPFC during one-back predicted post-training auditory dual-task performance, while neural activity changes in right DLPFC during 3-back predicted visual dual-task performance. The authors suggest that this neural activation may reflect improvement in central executive processing that could facilitate both working memory ability and dual-task coordination in older adults.

In another case study in line with Westerberg and Klingberg’s study in 2007—using WMT alongside fMRI—a 38-year old man with glycogen storage disease type IV and memory complaints was given CogMed training (61). He completed CogMed over 25 sessions, with each session consisting of eight verbal and visuospatial tasks, the difficulty adjusted based on the man’s daily performance over 25 weeks. The authors reported increased cortical activation in predominantly right frontoparietal regions 1–3 months after training, but both increased and decreased activation 6‐months later, which particularly corresponded to improvements in auditory working memory (remembering a story), and may again be an indication of improved, long-lasting cognitive updating ability associated with greater cortical activation.

The next fMRI WMT study was published more recently in 2019, examining the neural and behavioral effects of an adaptive online verbal WMT in healthy adults with a mean age of 56 years (62). The WMT—over 4 weeks—was based on the n-back task to the maximum 9-back level (identifying when the current auditory stimulus was the same as one presented 9-items previously). The authors reported that WMT was associated with decreased activity in fronto-parieto-cerebellar circuitry, as well as limbic regions, suggesting greater efficiency with decreased activity. The training effects also extended to improvements to other working memory tasks

In another fMRI study published in 2019 (63), the effects of a 5-week single-level n-back training on brain activation in a healthy population versus a no contact control group were examined. It was found that training improved working memory accuracy and response times associated with better neural efficiency (reduced activation) that persisted 5 weeks after training in frontal superior/middle cortex, inferior parietal cortex, anterior cingulate cortex, and middle temporal cortex. In another recent fMRI study, WMT with n-back levels 0–3 back was examined as an adjunct to stimulant medication for ADHD, compared to a non-active training group (46). The authors reported decreased neural activation as working memory load increased and when comparing baseline to end of treatment (48 sessions of 30 min), in the right insula, right putamen, left thalamus, and left pallidum, and increased activation in similar regions after a comparative sustained attention task. The authors conclude that changes in brain activity may precede behavioral modifications in working memory and sustained attention, but not in inhibitory control.

Another 2019 fMRI study examined the effects of CogMed WMT on neural structure and function of children (average age almost 8 years’ old) born extremely pre-term (<28 weeks’ gestational age) or born with extremely low birth weight (64). Children were given CogMed for 45‐min sessions, 5 days a week over 5–7 weeks and structural/functional MRI was measured at baseline and after training (versus placebo active control training). While the authors’ found no significant structural changes related to WMT, they reported increased neural activation in the precuneus and posterior cingulate cortices while completing the n-back task in the scanner after CogMed training. However, the authors’ concluded that these findings were not strong enough to conclude that CogMed led to significant neuroplasticity changes in children born pre-term or with extremely low birth weight.

Finally, in the most recent fMRI study (a randomized control trial), there is some evidence for the contribution of the catechol-O-methyltransferase (COMT) (Val-158/108-Met) polymorphism (rs4680) to WMT related prefrontal cortex plasticity (65). This COMT gene polymorphism results in a valine to methionine mutation, whereby three possible outcomes occur: homozygous met (met/met); homozygous Val (Val/Val) or heterozygous (Val/Met). It has been shown that the homozygous Val variant metabolizes dopamine at up to four times the rate of a polymorphism expressing the Met variant (either homo-or-heterozygous) in brain areas such as the prefrontal cortex (66). In the study by Zhao and collegues, WMT led to significantly reduced activation in the left prefrontal cortex, but especially for the Met (hetero – and homozygous) versus Val allele carriers, in that neural activation in the left prefrontal cortex of Val/Val carriers was unaltered between pre- and post-training.

In summary, the fMRI studies of WMT reveal decreased activation after WMT training in frontoparietal, cingulate, insular and occipital cortex, regions, possibly indicative of either practice effects or increased neural efficiency, for example, greater encoding efficiency over the time course of training. In addition, an inverted-U shape relationship may occur during WMT, with increased activation in the right inferior frontal and right intraparietal cortices after 2 weeks of increased load n-back training shown, but with reduced activation during consolidation and after a month of training. Furthermore, improved connectivity associated with WMT-related increased activation in middle frontal gyrus and inferior/superior parietal cortices may indicate greater functional connectivity between regions. Moreover, while the debate continues about whether reduced or increased activation signals greater neural efficiency following WMT, it is also suggested that wider recruitment of frontoparietal regions during the course of training (e.g., a maximum five weeks) is indicative of better neural functioning. Interestingly, pre-training striatal activation, and also older-age decline in striatal activation was shown to be related to reduce WMT transfer gains, and it appears that reduced activation in working memory-related brain regions most commonly reflects improved cognition, particularly in the left DLPFC, right superior frontal gyrus, bilateral supramarginal gyrus, bilateral middle temporal gyrus, and bilateral middle occipital lobe in adults. In addition, increased neural efficiency (reduced activation) after WMT in frontoparietal regions is typical of a “young versus older” response to training, with older adults’ WMT improvements related to more efficient DLPFC activation. Taken together, predominantly frontoparietal and striatal activation is associated with WMT benefits, but it is not clear whether increased or decreased activation is most significant, and how this differs in young versus older people.

#### Task-Based Functional Connectivity fMRI Studies

Three fMRI studies used functional connectivity analyses to examine how WMT might alter global and regional neural network activity—which is often universally consistent [e.g., default mode network, executive control network (ECN), salience network, dorsal attentional network (DAN), among others]—during fMRI cognitive task performance (67). In the first WMT functional connectivity study, Jolles and colleagues in 2011 (68) examined whether 6 weeks of adaptive WMT altered functional connectivity at rest, in 15 adults versus 9 children. The authors reported that in adults, increased functional connectivity was observed after verbal WMT, between the right medial prefrontal gyrus and other regions of the frontoparietal network, including bilateral superior frontal gyrus, paracingulate gyrus, and anterior cingulate cortex. Furthermore, children showed reduced functional connectivity between the medial prefrontal cortex and right posterior middle temporal gyrus. In addition, correlational analyses with performance changes revealed a positive link between performance increases and changes of frontoparietal connectivity, and a negative link between performance increases and changes of default network connectivity. The authors conclude that preparatory effects prior to cognitive task performance are represented in resting state networks and can be altered with training, although the degree of practice effects could not be ascertained. In a study by Sun and colleagues (69), graph theory analysis was applied to two participants who engaged in spatial n-back training for three consecutive days. The authors reported a significant decreased clustering coefficient and normalized shortest path length, suggesting a reduced local efficiency with an increased global efficiency after WM training. In another functional connectivity fMRI study of 20 WMT sessions, healthy participants were randomly assigned to either intensive dual n-back or a demanding visuospatial attention task (70). The authors reported that WMT significantly improved functional connectivity in two large-scale frontoparietal networks, namely, the ECN and the DAN. Furthermore, the magnitudes of increased functional connectivity, particularly in the DAN, correlated with cognitive intensity during the tasks and improved task performance. Thompson and colleagues conclude that these results provide insight into the adaptive neural systems that underlie large gains in working memory capacity through training.

In summary, functional connectivity studies of WMT implicate improvements (increased functional connectivity) within the ECN and dorsal attention network (DAN) in adults, but in children the ECN showed reduced connectivity following training, which may also be related to reduced reward processing in the younger group. Moreover, in an exploratory study of two participants, reduced local efficiency and increased global efficiency has been shown after WMT. These findings corroborate the task-based fMRI studies, in that both modalities implicate improvements in neural activation within prefrontal cortex networks following WMT, and while this does not demonstrate far-transfer (e.g., in relation to improvements in impulse control per se), it is worth remembering that better working memory performance is associated with reduced impulsivity (32, 33).

#### Resting State Functional Connectivity Studies

One pilot study of resting state fMRI (brain activation independent of task performance) used 25 sessions of 30- to 45-min CogMed training for children with neurofibromatosis type 1 (71). Neurofibromatosis is associated with significant learning and memory deficits. Following training, reduced fractional amplitude was found in the cerebellum and thalamus, and decreased regional homogeneity in the superior frontal sulcus, and increased regional homogeneity in the fusiform gyrus (a visual area). These functional brain changes corresponded to **s**ignificant post-training improvements on CogMed tasks: Identification task speed and Groton Maze Learning accuracy increased.

A more recent resting state fMRI study using WMT examined the effects of computer-based training on functional connectivity of attentional networks in primary school children (72). The children with a mean age of 9 years completed two sessions per week over 13 weeks. It was found that greater functional connectivity within the attentional networks (incorporating the anterior cingulate, anterior insula, lateral prefrontal cortices, and basal ganglia) occurred after WMT. In addition, stronger functional connectivity between a right middle frontal gyrus cluster and lateral parietal/superior temporal cortices corresponded to higher inhibitory control scores.

In summary, the resting state fMRI studies link to the previously summarized fMRI studies, in that neural changes following WMT tend to occur in lateral prefrontal, parietal, and basal ganglia regions, and in these two studies to date, it appears that increased resting state activation in the attentional networks is associated with greater working memory efficiency, whereas the task-based fMRI studies show both increased and decreased activation associated with cognitive efficiency.

### Structural Imaging Studies

Structural imaging techniques capture the tomography (e.g., volume, thickness, folding of gyri, and shape) of different regions of the brain, often through nuclear magnetic resonance which generates high resolution images (73). In six structural brain imaging studies (utilizing DTI, MRI, voxel-based morphometry), cortical thickness, volumetric, connectome, and white matter tractography differences have been reported after WMT. In the first structural study by Takeuchi and colleagues (74), the capacity of working memory, associated with integrity of white matter tracts in frontoparietal regions was examined using voxel-based morphometry and DTI of fractional ansiotrophy after 11 healthy participants completed: 1) Visuospatial, 2) Operational N-Back, and 3) Dual n-back WMT. The level of WMT achieved correlated with increased fractional ansiotrophy (a measure of white matter density) in white matter regions near to the intraparietal sulcus and the anterior corpus callosum after training, suggestive of neuroplasticity/myelination changes. Takeuchi and colleagues continued their investigations by conducting a structural brain imaging study of mental calculation and working memory in 55 healthy males and females with a mean age of 21.7 years. They examined the effect of WMT on gray matter volume using voxel-based morphometry (75). It was found that mental calculations improved verbal letter span and mental arithmetic but reduced levels of creativity, and these findings were in line with volumetric reductions in the bilateral fronto parietal regions and the left superior temporal gyrus. The authors concluded that volumetric reductions also demonstrate neuroplasticity changes, perhaps in relation to greater neural efficiency and synaptic pruning of disorganized connectivity within the brain.

In another structural study, Envig and colleagues examined the plasticity effect of WMT on cortical thickness and volume in 42 male and female participants who were on average 60 years of age, after 8 weeks of intensive training using memorization techniques of location sequences throughout a person’s house (76). It was found that cortical thickness changed after WMT in the right fusiform and lateral orbitofrontal cortex, which correlated positively with improvement in source memory performance. This suggests a possible functional significance of the structural changes that may be related to improved attentional control and visuospatial scratchpad maintenance memory enhancement.

Another structural study examined the dynamics of the human structural connectome underlying WMT (77). It was reported with graph theoretical analysis of the structural (white matter) network connectivity (“connectome”) that there was increased global integration within a frontoparietal attention network following adaptive WMT (40 sessions of 45-min CogMed) compared with the nonadaptive training group. Moreover, the authors state that increased efficiency of the frontoparietal network was best captured with connection strengths derived from MRI metrics that were more sensitive to differences in myelination than previously used diffusion (fractional anisotropy or fiber-tracking recovered streamlines).

In another structural brain imaging study of WMT using MRI and voxel-based morphometry, in-patients with chronic methamphetamine use who were abstinent for at least one week prior to the intervention, were given 20 half an hour sessions over 4 weeks of the C-Ya App (based on 0- to 3-back of the N-back task), and were scanned at baseline and after 4 weeks (33). Compared to a treatment as usual only group who were also scanned, the WMT group showed increased bilateral basal ganglia volume that corresponded to improvements in self-reported impulsivity and self-regulation. The authors suggest, in line with previous studies (43) that increased function of the striatum is associated with better working memory ability (e.g., updating of information, effective maintenance of cognitive strategies independent of external stimulation).

In another structural imaging study of adaptive WMT, white matter plasticity in the main frontoparietal tract, namely, the superior longitudinal fasciculus, compared to the cingulum bundle as a control, was measured in line with potential cognitive benefits (78). The authors reported that white matter diffusivity changes—reflecting better working memory capacity—were specifically observed in the right dorsolateral superior longitudinal fasciculus and the left parahippocampal cingulum. Specifically, the changes were shown as increases in R1, restricted volume fraction, fractional anisotropy, and reduced radial diffusivity. Interestingly, the opposite pattern of changes in white matter microstructure was observed in the non-adaptive control session. The authors conclude that the changes are consistent with new microstructural myelination following WMT.

In summary, structural brain imaging studies of WMT have demonstrated increased white matter density within the intraparietal sulcus and the anterior corpus callosum after training (potentially an indication of increased myelination/neuroplasticity). Other studies found volumetric reductions in the bilateral frontoparietal regions and left superior temporal gyrus, which may indicate synaptic pruning underlying greater neural efficiency. In addition, increased cortical thickness in the right fusiform and lateral orbitofrontal cortex have been observed in people with an average age of 60, suggesting improvements in attentional control. And in line with fMRI studies, connectome analyses of mictrostructural changes after WMT reveal increased global integration within a frontoparietal attention network, indicative of greater neuronal efficiency. Moreover, in line with some fMRI studies, increased bilateral basal ganglia volume after WMT in people treated for methamphetamine addiction is associated with improvements in impulsivity and self-regulation. Finally, white matter plasticity (e.g., diffusivity changes) in the main frontoparietal tract, namely, the superior longitudinal fasciculus, compared to the cingulum bundle as a control, was shown to occur following WMT. Thus, in conjunction with fMRI studies, structural imaging studies confirm the involvement of frontoparietal and striatal changes following WMT.

### Transcranial Direct Current Stimulation (tDCS)

This method applies low-level, continuous electrical currents delivered *via* electrodes across the scalp. Historically, tDCS was introduced to treat depression and other mood related conditions, but has since garnered attention due to some success delivering specific cognitive gains (79). To date, six studies have examined the effects of tDCS on neural activation during WMT. Mounting evidence suggests that tDCS can produce even greater gains than WMT alone, its mode of action consisting of applying a positive (anodal) or negative (cathodal) current *via* electrodes to an area of the brain within the working memory network (e.g., the dorsolateral prefrontal cortex) to facilitate the depolarization or hyperpolarization of neurons, respectively. In the first tDCS WMT study, Martin and colleagues gave 10 sessions of dual n-back training with active or sham tDCS versus tDCS only (80). In line with predictions, active tDCS enhanced participants’ working memory accuracy (but not skill acquisition) compared to those who received sham tDCS or active tDCS only. In a follow-up study, the same group examined the optimal timing of active tDCS to enhance WMT performance (81). Healthy participants received, in random order, 30 min of anodal tDCS to the left DLPFC immediately before (“offline” tDCS) and during performance (“online” tDCS) of a dual n-back WMT. The authors found that online tDCS was associated with most significant within-session greater skill acquisition and conclude that conducting WMT alongside active tDCS might provide greater cognitive gains than providing “offline” tDCS prior to the start of WMT.

In another tDCS study, authors’ examined whether stimulation at home during 5 sessions of WMT improved older adults’ cognition (82). It was found that 2 mA of tDCS induced significantly greater far transfer gains one month after training. Moreover, these cognitive gains were observed on far transfer tasks (subtract 2 Letter Span Task, automated Operation Span and a spatial and visual WM task), and stimulation was well tolerated by all participants. Of note, for the first time the authors also utilized functional near infra-red spectroscopy (fNIRS) to examine potential neural changes associated with WMT, but were unable to find conclusive evidence.

In another tDCS study by the same group a year later, the authors examined whether tDCS effectiveness is mediated by polymorphism in the Catel-O-methyl-transferase (COMT val158) gene (83). The authors found that those with the val/val COMT genotype gained most from 1.5 mA tDCS during visual WMT and from 1 mA tDCS during spatial WMT. For met/met polymorphisms, 2 mA resulted in significantly poorer performance compared to 1.5 mA on spatial WMT. The authors’ concluded that variations in COMT val158met may predict the nature of WM improvement after initial and longitudinal tDCS. It is important to note that in the previous fMRI study that examined the contribution of COMT polymorphisms to WMT-related neuroplasticity, it was conversely found that neural activation in the val/val polymorphism group was least influenced by training (65). The discrepancy between the two studies could be due to the differences associated with encouraging neuroplasticity effects *via* WMT, versus brief neurostimulation *via* tDCS. However, more studies need to be conducted to ascertain how COMT polymorphisms are linked to WMT and prefrontal cortex function.

In another tDCS study, Jones and colleagues attempted to better understand how tDCS creates cognitive gains reflected in brain function when coupled with WMT (84). To this aim, EEG recordings were taken before and after a week of visuospatial WM change detection training, during which participants completed four sessions of frontoparietal tDCS (active anodal or sham). It was found that those who had anodal tDCS experienced greater improvement on the WMT, compared to sham tDCS, and that this improvement was reflected in frontal-posterior alpha band power, and theta and low alpha oscillations associated with greater neural synchronization.

In the most recent tDCS study of WMT, the effects of neurostimulation on working memory function in older adults, with a mean age of 74 years, were examined (85). The study used N-back training (2-back versus 0-back) over 10 sessions and found that active versus sham tDCS produced significantly increased connectivity between the left DLPFC and right inferior parietal lobe. The authors suggest that this demonstrates a method to remediate cognitive decline in older age.

In summary the tDCS studies of WMT demonstrate that active tDCS appears to enhance participants’ working memory accuracy (but not skill acquisition). Moreover, online tDCS (e.g., given during WMT) appears to be associated with most significant skill acquisition, suggesting that conducting WMT alongside active tDCS might provide greater cognitive gains than providing “offline” tDCS (e.g., prior to the start of WMT). In addition, it appears that a greater level of stimulation (e.g., 2 mA tDCS) induces significantly greater far transfer gains. Genotype effects on the efficiency of tDCS during WMT show that the val/val versus met/met COMT genotype may gain the most from 1.5 mA tDCS during visual WMT and from 1 mA tDCS during spatial WMT. In contrast, for met/met polymorphisms, 2 mA may be related to poorer performance compared to 1.5 mA on spatial WMT. In terms of linking tDCS to neural activation, active tDCS improvement is linked to frontal-posterior alpha band activity, and theta and low alpha oscillations associated with greater neural synchronization. Similarly, in older adults, active versus sham tDCS increases connectivity between the left DLPFC and right inferior parietal lobe. As such, the tDCS studies appear to corroborate the findings of fMRI and structural studies, in that fronto-parietal activation is improved with stimulation.

### Electroencephalography (EEG)

This technique is a non-invasive, electrophysiological imaging modality which captures topographical electrical activity direct from the cortex through electrodes on the scalp (86). Three event-related potential studies using EEG have also been conducted to examine how WMT improves connectivity across brain networks. In an EEG study by Kunda and colleagues, transcranial magnetic stimulation (TMS) was used to strengthen effective connectivity—the extent to which one node or region of the cerebrum acts in tandem with another—in 30 healthy males and females. It was shown that WMT benefits are transferred to other cognitive tasks in association with more efficient frontoparietal and parieto-occipital network connectivity (87). The authors go on to state that WMT (and TMS) evokes greater efficiency within these networks to support better stimulus processing, as demonstrated by alterations in EEG individual differences associated with greater short-term memory capacity and visual search performance.

In another EEG study by Oelhafen and colleagues (88), healthy young adults were trained for three weeks with a high or low interference training version of the dual n-back task versus a passive control group. The authors’ found that training transferred to an attentional test, and that this was reflected in increased P300 activation (associated with greater selective attention) in the parietal cortex during the high interference training only. Oelhafen and colleagues concluded that WMT with an interference component may have a significant effect on altering brain processes than standard low/no interference WMT.

In the latest EEG event related study of WMT using dynamic causal modeling to examine effective connectivity changes, Chen and colleagues contrasted P300 with N160 amplitudes—the former associated with selective attention, the latter associated with detection of novelty in review and inhibitory control (89). The authors gave participants training with either a 3-back visual WMT, or a repetition practice task, and found that visual WM training altered the frontal-parietal networks, namely, the ECN and the DAN. In contrast, the repetitive practice task modulated the parietal-frontal connections underpinning P300 activation for selective attention. The authors concluded that they were able to pinpoint specific neuroplasticity changes associated with WMT.

EEG studies, in summary, have demonstrated that WMT benefits are transferred to other cognitive tasks in association with more efficient frontoparietal and parieto-occipital network connectivity—particularly the ECN and DAN—in line with findings from other neuroimaging modalities.

### Cerebral Perfusion

This measurement examines the net pressure gradient leading to cerebral blood flow in the brain. One study has examined significant training effects after seven days of n-back WMT compared to an active control group in relation to changes in cerebral blood perfusion (90). WMT was associated with both training gains and cross-task transfer compared to the active control group. Moreover, the authors reported increased perfusion during WMT in selected regions, suggesting strategies employed to cope with high task demand. Additionally, the authors reported increased blood flow at rest in regions where training effects were apparent, and that rest perfusion correlated with task proficiency, suggestive of improved neural readiness to engage in the task.

### Neurogenetic Analyses

Neurogenetic analyses examine how genetic variation, including genetic mutation, affects behavior and cognition. Studies have shown that a genetic variation linked to increased dopamine metabolism in the prefrontal cortex (catechol-O-methyltransferase Val158Met; COMT) amplifies age-related alterations in working memory performance. Furthermore, in younger participants, the influence of these dopaminergic genetic polymorphisms is suggested to increase as working memory load increases. On this basis, a recent fMRI study by Heinzel and colleagues compared younger and older adults’ neural responses during 12 sessions of increasingly difficult adaptive n-back WMT (17). Firstly, younger adults demonstrated greater behavioral gains than older adults after WMT. Furthermore, this age-related difference in effective WMT performance was associated with decreased plasticity in older adults carrying the Val/Val COMT genotype, with no significant genetic interaction observed in younger adults. This suggests that prefrontal cortex dopaminergic synaptic plasticity associated with WMT is significantly influenced by COMT polymorphisms, particularly in older adults. Similarly, in the fMRI study by Zhao and collegues, WMT led to significantly reduced activation in the left prefrontal cortex, but especially for the Met (hetero – and homozygous) versus Val allele carriers, in that neural activation in the left prefrontal cortex of Val/Val carriers was unaltered between pre- and post-training (65). Conversely, the tDCS study examining the influence of COMT showed that those with the val/val COMT genotype gained most from 1.5 mA tDCS during visual WMT and from 1 mA tDCS during spatial WMT. For met/met polymorphisms, 2 mA resulted in significantly poorer performance compared to 1.5 mA on spatial WMT. However, more studies examining the link between genetic polymorphisms and neural far-transfer effects during WMT are needed before definitive conclusions can be made.

## Discussion

### Explanation of Findings

The first aim of this review has been met, to demonstrate that brain imaging studies of WMT report significant neural effects often independently of behavioral changes in frontoparietal and frontostriatal circuitry. This review has also met its second aim, to demonstrate that there is a paucity of far-transfer measures of impulse control in neuroimaging studies of WMT, despite a wealth of evidence emphasizing that frontoparietal and striatal circuits not only contribute to changes in working memory function across the lifespan but are also associated with the cognitive control of impulsivity (34, 91). As such, studies examining the neural effects of WMT associated with impulse control could be a fruitful research avenue to pursue for those aiming to improve neural processes in people with addictive disorders, which often demonstrate comorbid impulse control deficits (91). This review has also highlighted that the majority of brain imaging studies of WMT have relied on fMRI—including task-based, resting state and functional connectivity analyses (with neural links to polymorphisms in the COMT *val/val* genotype). Other imaging studies have utilized structural measures, including MRI, voxel-based morphometry for volumetric analyses, shape and cortical thickness measures, and examination of myelination neuroplasticity changes in the “connectome” after WMT. In addition, some fewer studies have begun to explore the utility of brain stimulation measures (e.g., tDCS and TMS) as an adjunct to improve WMT far-transfer effects that may translate into long-term behavioral changes. Other neuroimaging studies have used EEG and cerebral blood perfusion measures. Finally, the studies in this review have highlighted three debates within the WMT neuroimaging field: a) the pattern of brain activation underlying far-transfer, b) whether WMT is associated with increased or decreased neural activation, and c) whether there are differential neural changes with WMT in younger versus older participants.

### Considering the Debates

#### Far-Transfer to Other Cognitive Tasks and Changes in Brain Regions

Various neuroimaging studies reported—alongside neural changes—far transfer effects of WMT to other un-related cognitive domains (20, 43, 56, 60, 61, 75, 82, 87). Using fMRI, Dahlin and colleagues demonstrated significant transfer gains of an updating task (memorizing letter sequences) to working memory performance on the n-back task that was mediated by pre-training striatal activation. Takeuchi and colleagues used structural MRI to show that performance of an intensive adaptive training of working memory using mental calculations (IATWMMC) enabled transfer gains to verbal letter span and complex arithmetic ability, but deteriorated creativity, in line with reduced brain volume in bilateral fronto-parietal regions and the left superior temporal gyrus. Schneiders and colleagues showed transfer effects for auditory but not visual WMT that corresponded to decreased activation in the right inferior frontal gyrus and right inferior parietal cortex, reflecting less demand on general attentional control processes. Kundu and colleagues found transfer gains of increasing load n-back training to short-term memory and attention processes (visual search performance on Tetris), using EEG measures that highlighted greater effective connectivity in frontoparietal and parieto-occipital networks. Heinzel and colleagues showed—with fMRI—that 12 sessions of 45-min adaptive n-back training improved performance on a delayed recognition task (Sternberg) as well as behavioral transfer to executive functioning, processing speed, and fluid intelligence, and that this transfer gain was reflected in decreased right lateral middle frontal gyrus/caudal superior frontal sulcus (Brodmann area, BA 6/8) activation. In a later fMRI study by Heinzel and colleagues, they examined whether single-task WMT alterations in neural activity might support performance in a dual-task setting. No transfer to single-task performance was found. However, costs for audio-visual dual tasks decreased at post-measurement after WMT. These cost improvements were reflected in bilateral DLPFC activation changes. In a study by Stephens and Berryhill, it was demonstrated that 2Ma of tDCS led to greater WMT transfer gains on unrelated tasks for older participants at home one month after training had ceased.

#### Increased or Decreased Neural Activation After WMT

The majority of fMRI studies have reported decreased activation following WMT (40–43, 46, 54, 56–58, 60, 63). Garavan and colleagues examined neural responses after WMT in five healthy participants using a delayed match-to-sample task and reported that reduced activation appears to be a consequence of practice effects. (40). However, subsequent fMRI studies over the next two decades have honed alternative explanations; such as an inverted U- shape (41) or sigmoid (59) pattern of activation, as WMT cognitive load increases (58). For example, Hempel and colleagues reported an inverse U-shaped quadratic function with a negative exponent that was significant in the right intraparietal sulcus/superior parietal lobe under the 1-back and 2-back conditions only: during training there was increased activation, whereas at the beginning and end of the training/consolidation period these regions showed reduced activation. These non-linear activation patterns—Hempel and colleagues argue—are reflective of a mediation effect of differential neuronal mechanisms during a memory-demanding task, including: a) repetition suppression, b) enhancement, and c) delay activity (92). The authors go on to explain a theory that the prefrontal and the parietal networks may have parallel functions in WMT; a stimulus and memory enhancement mechanism associated with the saliency network and prefrontal cortex activation, and an active information suppression mechanism that occurs non-consciously during stimulus repetition. These parallel neural mechanisms may explain increases and decreases in activation according to the duration and load of WMT.

Other fMRI studies have reported increased activation following WMT (43, 53, 55, 58, 61, 64). Olesen and colleagues used 3 CogMed tasks in a small sample of 8 healthy participants to evoke increased frontoparietal activations five weeks after training. Westerberg and Klingberg—again using CogMed—showed in three participants that the extent of activated cortical area within the middle and inferior frontal gyri corresponded to the extent of working memory improvements following training. Dahlin and colleagues linked increased striatal activation prior to training, to near-transfer gains. Schweizer and colleagues used a 5-week affective dual n-back training to demonstrate improved cognitive control of emotional responses, corresponding to greater activation in the frontoparietal network and subgenal anterior cingulate (an area associated with affect regulation). Finally, Lee and colleagues examined a 38-year old man—using fMRI—with glycogen storage disease type IV and memory deficits, and found that WMT improved his memory in line with increased right frontoparietal activation; however, there were both increases and decreases in this network 6-months’ later corresponding to auditory working memory ability and efficient story recollection.

The significance of increased striatal activation and plasticity in the role of WMT transfer and effective training consolidation was particularly highlighted by two studies (one functional, one structural MRI). Dahlin and colleagues conducted an fMRI study to examine whether overlapping neural patterns could predict the degree of transfer to an untrained cognitive task. They used an updating task (remembering letter sequences) to demonstrate significant transfer gains to the 3-back level of the n-back task (but not to the non-updating Stroop task), the success of which was mediated by pre-training striatal activation, which was not present in older adults (43). The authors concluded that the ability to dynamically manipulate and update information during incoming stimulus processing (a key feature of working memory) was significantly associated with striatal dopaminergic gating function, a form of synaptic gating where dopamine is suppressed or facilitated in the striatum. Similarly, in a study of chronic methamphetamine users currently abstinent and engaging in cognitive-behavioral therapy, Brooks and colleagues demonstrated that 4 weeks (20 half-hour sessions) of 0-3-back WMT led to increased bilateral basal ganglia volume (incorporating the striatum) in line with improved self-reported impulse control and self-regulation (33). In line with Dahlin and colleagues’ hypotheses (43) as well as others (34, 49) increased basal ganglia volume might indicate that WMT forges neuroplasticity changes, enabling more efficient updating capabilities, which support new cognitive strategies for better cognitive control of impulsivity. In line with the importance of striatal structural and functional changes after WMT, Nemmi and colleagues have recently tested the extent to which dopamine-related genes (e.g., COMT and DAT-1), striatal activation and morphology have been associated with increased working memory capacity in different ages of adolescents (93). The authors measured working memory in adolescents twice: at age 14 and 19 years and also took neural and genetic measures. They found a significant interaction between putamen size and DAT1/SLC6A3 rs40184 polymorphism, specifically that TC heterozygotes with a larger putamen at age 14 showed greater working memory capacity at age 19. The authors go on to explain that the effect of the dopamine transporter gene (DAT- 1) polymorphism on the beneficial development of working memory was related to changes in striatal morphology. That said, given the paucity of WMT studies examining neural changes to striatal structure and function, it is too early to comment on hypotheses about WMT far transfer to improvements in impulse control, particularly in those with addictive disorders.

#### Neural Processes of WMT in Young Versus Older Participants

Neuroimaging studies have shown that WMT has differential effects in younger versus older participants (17, 20, 43, 46, 59, 69, 71, 82). Dahlin and colleagues suggest a neural process underlying the often reported finding that working memory capacity and efficiency worsens with age, whereby pre-training striatal function enables updating transfer gains in older people during working memory (43). Similarly, Heinzel and colleagues show that increasing-load n-back WMT enables older adults to have “youth-like” increased frontoparietal activation, with reduced activation at lower cognitive loads reflected greater neural efficacy in older participants (59). Moreover, the authors have shown that a genetic variation associated with increased dopamine metabolism in the prefrontal cortex (Catechol-O-methyltransferase: Val158Met; COMT) amplifies age-related working memory decline, in terms of decreased behavioral plasticity in older (but not younger) adults carrying the *Val/Val* genotype (17). Comparing one young adult and one older adult (32 versus 60 years of age), Sun and colleagues demonstrated functional connectivity differences using graph theory analyses (69). In both the young and older participants, improved working memory was associated with reduced local efficiency and an increased global efficiency, although the younger participant reached higher levels on the n-back task.

In line with other structural and functional connectivity studies, Sun and colleagues argue that WMT, from just a few days to months, can augment the human brain connectome, *via* synaptic plasticity and myelination changes (68, 70, 74, 75). Moreover, a tDCS study showed that greater far-transfer WMT gains could be achieved in older people using tDCS at the 2Ma frequency at home (82). Thus, while working memory capacity and efficacy is known to decline as we age, in line with degeneration within frontoparietal and frontostriatal circuits, WMT may help to delay or slow this process. However, more research is needed in this area, particularly in terms of reversing neurodegeneration associated with chronic ageing and other degenerative conditions such as chronic stimulant use disorder.

### Limitations and Implications for Future Research

This review of WMT in neuroimaging studies was hampered by the variance in sample sizes across studies, ranging from one to fifty-five participants, with the majority reporting underpowered samples. In addition, while it is useful to review the neural effects of WMT from the perspective of different imaging modalities, the majority of studies utilized fMRI with considerable variation of methods (e.g., resting state, event-related, task-based block design, and functional connectivity). As such, it is difficult to form a clear conclusion as to the consensus in the field, or to conduct a useful meta-analysis of WMT brain imaging studies—which will need to be reserved for such a time that a sufficient number of comparable studies can be analyzed with Activation Likelihood Estimation (ALE) for example (94). Nevertheless, collectively, studies using fMRI, EEG, tDCS, MRI, and DTI have contributed a significant amount over the last two decades, to the debate on how WMT alters brain processes in healthy young and older adults. Given that a major strength of this review is in highlighting that various imaging modalities have replicated effects in frontoparietal and frontostriatal circuitry—these data can be used to inform WMT interventions for addictive disorders that often demonstrate dysregulation in similar brain circuitry.

## Conclusion

The neuroimaging studies reviewed highlight a potential non-linear relationship between brain changes and far-transfer effects that may be shrouding the measurement of significant WMT benefits. Imaging studies show that training load, duration, modality, participant age, and even participant genotype can influence the effects of WMT on the brain. In addition, neural changes may only occur gradually and prior to the emergence of significant behavioral changes, hinting at the need for researchers interested in this field to control the impulse to throw the baby out with the bathwater, and to pursue other measures. This may be especially true when considering research into the neural effects of WMT for those with addictive disorders, whose enduring impulse control deficits tend to increase the likelihood of relapse. WMT is a “do-no-harm” intervention that—according to the studies reviewed here—may improve impulse control deficits associated with aberrant frontoparietal and striatal processes in people with addictive disorders.

## Author Contributions

SB conceived and wrote the manuscript. RM-P created the table of studies. JT helped to substantially revise the manuscript. HS helped to revise the manuscript.

## Conflict of Interest 

The authors declare that the research was conducted in the absence of any commercial or financial relationships that could be construed as a potential conflict of interest.

## Supplementary Material

The Supplementary Material for this article can be found online at: https://www.frontiersin.org/articles/10.3389/fpsyt.2020.512761/full#supplementary-material
