# Supplementary material for: Review of the Neural Processes of Working Memory Training: Controlling the Impulse to Throw the Baby Out With the Bathwater
Source: Front Psychiatry. 2020 Sep 10;11:512761. doi: 10.3389/fpsyt.2020.512761 (PMC7511702; doi:10.3389/fpsyt.2020.512761)
Supplement: Supplementary file 1 [file Table_1.docx]

| **Author** | **Title** | **Working Memory Training** | **Duration** | **Sample** | **Method** | **Main Findings** |
| --- | --- | --- | --- | --- | --- | --- |
| **fMRI studies – task based (n=20)** | | | | | | |
| **Garavan et al 2000** | Practice-related functional activation changes in a working memory task | Delayed match-to-sample visuospatial working memory VSWM on moderate or extensive task (Jonides et al 1993). Series of dots presented on screen in scanner, 50% encircled – participants indicated circle. 44 runs of the VSWM task  (i.e., 880 trials) and were scanned while performing runs  1–4, runs 21–24, and runs 41–44. Remaining runs were  practiced in isolation in a quiet room | One session of practice prior to scanning | Five right-handed participants (mean  age 28; two female) | fMRI 1.5T | Performance analyses revealed improved response times, but not accuracy, following relatively brief (Experiment 1) and lengthy (Experiment 2) periods of practice on a VSWM task. Activations in frontal (inferior, middle, and precentral gyri and superior frontal sulcus), parietal (intra-parietal sulcus, inferior parietal lobule, and precuneus) and cingulate (anterior and posterior) regions were observed as were bilateral insular and occipital activations. With the exception of the posterior cingulate, practice produced activation decreases in these regions, thus providing little evidence for  a re-organization of the functional neuroanatomy. |
| **Hempel et al 2004** | Plasticity of Cortical Activation Related to Working Memory During Training | *n*-back task - three conditions (0-back, 1-back, and 2-back, respectively) with increasing difficulty, each consisting of 16 stimuli presented  in a randomized a (45-second duration). | Twice daily training for four weeks. | Nine right-handed healthy male subjects (ages 26 to 32 years,  with 4 to 6 years of university-level education) | fMRI 1.5T | Inverted u-shaped function in mainly comparable frontal and parietal regions during a more intense training of a spatial n-back task. |
| **Landau et al 2004** | A functional MRI study of the influence of practice on component  processes of working memory | A face working memory task, one practice session during scanning. Presented with series of four intact and scrambled faces during encoding phase. At the retrieval phase participants were presented with a single face and had to decide if it was among the previous array of faces. | One practice session during scanning | Ten right-handed healthy participants (age range 22–27) | fMRI 1.5T | Practice-related decreases in fMRI signal and effects of memory load occurring primarily during encoding. This suggests that practice improves encoding efficiency, especially at higher memory loads. |
| **Olesen et al 2004** | Increased prefrontal and parietal activity after training of working memory | Three visuo-spatial working memory tasks were trained: Grid, Grid rotation and 3D Grid (Cogmed Cognitive Medical  Systems) A probe circle with a number in it  (1–5) appeared, and the task was to indicate whether the probe was in the  same location as any of the cues and, if so, whether the number corresponded  to the serial position of that cue. | Trained on the three tasks for five weeks | Experiment 1: Three male, right-handed volunteers (20–23 years of age)  Experiment 2: Eight healthy volunteers (29.3 ± 2.1 years of age; six females; one left  handed) | fMRI 1.5T | Increased activation in middle frontal gyrus and superior and inferior parietal cortices. |
| **Sayala et al 2006** | Increased Neural Efficiency with  Repeated Performance of a  Working Memory Task is  Information-type Dependent | Each run contained WM trials of a single type, either object or spatial  WM but not both within a single run. There were two conditions, each consisting of a different sequence of runs and classified according to which WM task was being  repeated: (i) ‘object repetition’ and (ii) ‘spatial repetition’. | One session of repeated runs of object/spatittasks. | 10 healthy participants (four females), aged 20--34 years | fMRI 3T | Decreases in neural activation in both spatial and object-selective brain areas (e.g. frontal, parietal, insular cortices) after spatial WM task repetition (and not the object-recognition task) that was independent of behavioural performance |
| **Westerberg & Klingberg 2007** | Changes in cortical activity after training of working  memory — a single-subject analysis | Tasks included a visuospatial WM task, a Span-Board Task, Stroop Task. | Training four to six days a  week for five weeks (20, 24 and 30 number of days for the three  subjects AP, DH and IK respectively) | Three healthy, male, right-handed volunteers (AP, DH, IK), aged 23, 20 and 22 years, participated in the training. A control group consisting of eleven healthy adult subjects, five men and  six women, mean age 25.8 SEM 1.5 years, undertook repeated  testing of the neuopsychological test battery with a five week test–retest period to provide a baseline for comparing the test–  retest improvements from the other subjects. | fMRI 1.5T | Increased in the middle and inferior frontal gyrus. The changes in activity were not due to activations of any additional area that was not activated before training. Instead, the changes could  be described by small increases in the extent of the area of activated cortex. |
| **Dahlin et al., 2008** | Transfer of Learning After Updating Training Mediated by the Striatum | Memory tasks: participants would be asked to recall the last four items from five different lists of previously presented stimuli (numbers, letters, colours, spatial locations). | 3 x 45-minute sessions per week for five weeks. | 24 young healthy right-handed participants (aged 20-31) randomly assigned to control or training groups (experiment 1). 22 healthy older adults (aged 65-71) randomly assigned to training and control groups (experiment 2) | fMRI 1.5 | After five weeks of updating training there was a transfer effect to a 3-back working memory test.  The transfer effect was based on a  joint training-related activity increase for the criterion (letter memory) and transfer tasks in a  striatal region that also was recruited pretraining. There was no transfer effect for the tasks not involve updating in the striatal areas. Changes in the striatum due to age limited the transfer effects of training. This suggests that if the trained task and unstrained task share overlap in processing components and neural correlates (e.g., updating) then transfer to the unstrained task is possible. |
| **Schneiders et al 2011** | Separating Intra-Modal and Across-Modal Training Effects in Visual Working Memory: An fMRI Investigation | An adaptive n-back paradigm, which was adapted from Jaeggi et al. (2008). In the n-back task, a series of stimuli  are presented consecutively and participants have to decide whether the present stimulus matches the stimulus that was presented positions back in the sequence. | 10 x 50-minute sessions over two weeks | Forty-eight undergraduate and graduate students,  26 females and 22 males, mean age = 23.67 years (age range = 19--31  years) | fMRI 1.5T | Larger training gains after visual working memory training compared with auditory or no training on a visual 2-back task. These effects were accompanied by specific training-related decreases in the right middle frontal gyrus arising from visual training only. Likewise, visual and auditory training led to decreased activations in the superior portion of the right middle frontal gyrus and the right posterior parietal lobule. |
| **Schneiders et al, 2012** | [The impact of auditory working memory training on the fronto-parietal working memory network](https://www.ncbi.nlm.nih.gov/pubmed/22701418) | Adaptive *n*-back paradigm | 8 x 50-minute auditory training sessions over 2 weeks | 16 healthy students, mean age: 21.13 years (17 females) | fMRI 3T | Transfer effects were found for the auditory but not for the visual transfer task. Decreased activation after training was found in the right inferior frontal gyrus, which may indicate increased neural efficiency in auditory working memory processes. The study also found decreases in in the right inferior parietal lobule reflecting less demand on general attentional control processes. |
| **Schweizer et al 2013** | Training the Emotional Brain: Improving Affective Control through Emotional Working Memory Training | Emotional working memory task (eWM) – n-back variation containing emotional stimuli | 20 (daily) x 20-30-minute sessions | 34 participants (20 female); age: 23 ± 2.4, mean ± SD). 15 assigned to placebo, 17 assigned to training, | fMRI 3T | Improvements on eWM performance in eWM trained but not placebo group. Transfer effects of training benefits to measures of cognitive control-emotional regulation. Behavioural changes associated with increase in BOLD signal activation in lateral prefrontal cortex, inferior parietal cortex and orbitofrontal cortex in trained compared to placebo group. eWM training may improve efficiency of the frontoparietal network. |
| **Buschkuehl et al, 2014** | [Neural effects of short-term training on working memory.](https://www.ncbi.nlm.nih.gov/pubmed/24496717) | Visuospatial *n*-back paradigm | 7 x 25-minute consecutive daily sessions | 55 participants (20 women; mean age: 21.8 years  Experimental group: 27 participants (10 women; mean age: 22.3 years)  Control group: 28 participants (10 women; mean age: 21.2 years) | fMRI | Results revealed that training on an N-Back task led to improved performance on the trained task. The experimental group also demonstrated cross-modal transfer, compared with an active control group. Increased perfusion during task performance was observed in selected brain regions, reflecting a neural response to cope with high task demand. Increased blood flow at rest in regions where training effects occurred was also found. |
| **Heinzel et al, 2014** | [Working memory load-dependent brain response predicts behavioral training gains in older adults.](https://www.ncbi.nlm.nih.gov/pubmed/24453314) | Adaptive *n*-back paradigm | 12 x 45-minute sessions over four weeks | 19 older participants (6 females, mean age: 66.0 years) and 18 younger adults (8 females, mean 24.1 years). | fMRI 3T | At low difficulty levels, decreases in BOLD FMRI responses were found after WM training. Working memory may improve neural efficiency in older adults |
| **Thompson et al, 2016** | [Intensive Working Memory Training Produces Functional Changes in Large-scale Frontoparietal Networks.](https://www.ncbi.nlm.nih.gov/pubmed/26741799) | Adaptive dual n-back paradigm with visual and auditory stimuli | 20 days | 39 healthy participants in active training group; between 18 and 45 yoa, R handed, good health and not taking psychoactive medication | fMRI 3T | Training resulted in task-specific expansion of dual N-Back abilities. Training differentially affected activations in two large-scale frontoparietal networks thought to underlie working memory: the executive control network and the dorsal attention network. Activations in both networks linearly scaled with working memory load before training, but training dissociated the role of the two networks and eliminated this relationship in the executive control network. Load-dependent functional connectivity both within and between these two networks increased following training, and the magnitudes of increased connectivity were positively correlated with improvements in task performance. These results provide insight into the adaptive neural systems that underlie large gains in working memory capacity through training. |
| **Heinzel et al, 2016** | Neural correlates of training and transfer effects in working memory in older adults. | *n*-back paradigm | 12 sessions over four weeks, 3 x 45-minute sessions per week. | 32 healthy older participants (60-75 years) – 16 assigned to control group and 16 assigned to training group. | fMRI 3T | WM performance improved with training and behavioral transfer to tests measuring executive functions, processing speed, and fluid intelligence was found. MRI findings indicate a training-related increase in processing efficiency of WM networks, potentially related to the process of WM updating. Performance gains in untrained tasks suggest that transfer to other cognitive tasks remains possible in aging. |
| **Heinzel et al 2017** | Transfer Effects to a Multimodal Dual-Task after Working Memory Training and Associated Neural Correlates in Older Adults - A Pilot Study. | *n*-back paradigm | 12 x 45-minute sessions over four weeks | 34 healthy older adults (age range 60-72). 18 included in training group (11 females; mean ± SD age = 65.78 ± 3.04), 16 matched participants included in control group (11 females, mean ± SD age = 65.00 ± 3.67). | fMRI 3T | *n*-back training lead to improved performance on trained task as well as a transfer to performance on a dual task (auditory and visual), but not single task, completed pre- and post-training. Reduced BOLD-activation in left DLPFC for one-back from pre- to post-testing; this predicted dual-task costs in the auditory task post-training. Changes in right DLPFC activation during three-back were associated with dual-task costs in the visual task after training. Suggests DLPFC activity decrease/increase during low/high WM load, respectively, may support dual-task performance. |
| **Lee et al 2017** | Neural correlates of adaptive working memory training in a glycogen storage disease type-IV patient | Adaptive WMT with CogMed – verbal and visuospatial working memory tasks | 50 sessions over 6 months | 38-year old male with diagnosis of glycogen storage disease type IV | fMRI 3T | Improvements on both trained and untrained working memory tasks up to 6‐months post-training. fMRI showed BOLD activation, particularly in left middle frontal gyrus–3 months after training, however, increases and decreases in activation were seen 6‐months later. WMT may be beneficial to patients with glycogen storage disease although ongoing training may be necessary for continued improvement. |
| **Emch et al., 2019** | Neural and behavioural effects of an adaptive online verbal working memory training in healthy middle-aged adults | N-back task, up to 9-back. | 32 training sessions, 4 sessions per week (8 in total) on personal computers via Inquisit. | 57 healthy participants (mean age 56, 28 male, 29 female) | fMRI 3T | Training resulted in an activity decrease in regions known to be involved in verbal WM (i.e., fronto-parieto-cerebellar circuitry and subcortical regions), indicating that the brain became potentially more efficient after the training. These activation decreases were associated with a significant performance improvement in the n-back task inside the scanner reflecting considerable practice effects. In addition, there were training-associated direct effects in the additional, external verbal WM task (i.e., HAWIE-R digit span forward task), and indicating that the training generally improved performance in this cognitive domain. These results led us to conclude that even at advanced age cognitive training can improve WM capacity and increase neural efficiency in specific regions or networks. |
| **Miro-Padilla et al., 2019** | Long-term effects of N-back training: an fMRI study | *n*-back paradigm - 1-back, 2-back and 3-back | 5 weeks (a total of 200 minutes) | 52 right-handed participants (21 male) aged between 21-16 years | fMRI 1.5T | n-back training improved performance in terms of accuracy and response speed in the trained group compared to the control group. These behavioral changes in trained participants were associated with decreased activation in various brain areas related to working memory, specifically the frontal superior/middle cortex, inferior parietal cortex, anterior cingulate cortex, and middle temporal cortex. Five weeks after training, the behavioral and brain changes remained stable. |
| **Rosa et al., 2019** | Effects of computerised cognitive training as add-on treatment to stimulants in ADHD: a pilot fMRI study | *n*-back with ACTIVATE™ - using 1,2 and 3-back. | 48 x 30-minute sessions | 20 ADHD subjects both genders all medicated with stimulants, aged 9-13 years of age, with IQ>80 | fMRI 3T | In N-back, decreases were observed in the BOLD signal change from baseline to endpoint with increasing WM load in the right insula, right putamen, left thalamus and left pallidum in the CT compared to the non-active group |
| **Zhao et al., 2020** | Evidence for the contribution of COMT gene Val158/108Met polymorphism (rs4680) to working memory training related prefrontal plasticity | *Visual-spatial span working memory task* | 20 sessions over 4 weeks | 60 Undergraduate students randomly assigned to adaptive training group (n=30) or the active control group (n=30) | fMRI 3T | This study provided evidence for the neural effect of a visual–spatial span training and suggested that genetic factors such as the *COMT* Val158/108Met polymorphism may have to be considered in future studies of such training. |
| **fMRI studies – resting state (n=2)** | | | | | | |
| **Yoncheva et al., 2018** | Computerized cognitive training for children with neurofibromatosis type 1: a pilot resting-state fMRI study | CogMed training | 25 x 30-45 minute sessions over 6-10 weeks | 16 children and adolescents (9 males, 7 females) with neurofibromatosis, aged 8-15 years | fMRI 3T | Pre- and post-training differences in resting state fMRI measures: decreased fALFF in the left cerebellum I-IV extending medially, the right cerebellum V, and bilateral thalamus; decreased ReHo in right superior frontal sulcus, and increased ReHo in a visual region (left occipital fusiform gyrus).  Significant post-training improvement on Cogstate tasks: Identification task speed and Groton Maze Learning accuracy increased |
| **Sanchez-Perez et al., 2019** | Computer-based cognitive training improves brain functional connectivity in the attentional networks: A study with primary school-aged children | Maths training and WM training | 2 weekly 30 minute sessions over 13 weeks | 33 children in the training group (19 boys, 14 girls, mean age 9 yrs); and 23 children in the control group (15 boys, 8 girls, mean age 9 yrs). | fMRI 1.5T | Brain functional connectivity changes were observed within the attentional networks (ATN), linked to improvements in inhibitory control. Findings showed stronger relationships between inhibitory control scores and functional connectivity in a right middle frontal gyrus (MFG) cluster in trained children compared to children from the control group. Seed-based analyses revealed that connectivity between the r-MFG and homolateral parietal and superior temporal areas were more strongly related to inhibitory control in trained children compared to the control group. These findings highlight the relevance of computer-based cognitive training, integrated in real-life school environments, in boosting cognitive/academic performance and brain functional connectivity. |
| **Structural brain imaging studies (MRI/DTI) (n=3)** | | | | | | |
| **Nordvik et al. 2012** | Exploring the relationship between white matter  microstructure and working memory functioning following  stroke: a single case study of computerized cognitive training | Neuropsychonline.com and CogMed QM - Visuospatial skills, attention, memory, executive functions (focusing on abstract categorization) and problem solving. | 1115 min (3-4 60-minute sessions per week, plus weekly session with clinical psychologist) | 1 male stroke patient (60y) | MRI 1.5T/DTI | Increased working memory, increased white matter integrity in anterior left and right hemisphere fractional ansiotrophy, and posterior left hemisphere fractional ansiotrophy which was linked to improved working memory. |
| **Brooks et al., 2016** | Psychological intervention with working memory training increases basal ganglia volume: A VBM study of inpatient treatment for methamphetamine use | C-Ya – variation of n-back paradigm (with distracting peripheral mosaic) | 5 x 30-minute (5 x 5-minute blocks, 1-minute break between blocks) sessions per week for four weeks | 66 male participants aged 18-50. 41 methamphetamine use disorder inpatients, 24 healthy matched controls | MRI 1.5T | Treatment as usual was associated with increased bilateral striatal volume, whereas those who engaged in WMT had more widespread bilateral basal ganglia volume increase, extending to the amygdala and hippocampus. Reduced bilateral cerebellar volume was associated with reduced impulsivity scores. |
| **Metzler-Baddeley et al 2017** | Dynamics of White Matter Plasticity Underlying Working Memory Training: Multimodal Evidence from Diffusion MRI and Relaxometry | Verbal and spatial span tasks (CogMed) | 5 sessions per week for 8 weeks (40 sessions/30 hours). | 40 healthy adults, 19-40 years of age, assigned to adaptive WMT (n=20, 11 females, 9 males) or non-adaptive control (n=20, 10 females, 10 males) groups. | DTI 3T | Adaptive training was associated with benefits in a “WM capacity” component and increases in a microstructural component (increases in R1, restricted volume fraction, fractional anisotropy, and reduced radial diffusivity) that predominantly loaded on changes in the right dorsolateral superior longitudinal fasciculus and the left para-hippocampal cingulum. In contrast, nonadaptive comparison activities were associated with the opposite pattern of reductions in WM capacity and microstructure. No group differences were observed for the myelin water fraction metric suggesting that R1 was a more sensitive “myelin” index. These results demonstrate task complexity and location-specific white matter microstructural changes that are consistent with tissue alterations underlying myelination in response to training. |
| **Transcranial direct current stimulation (tDCS) studies (n=6)** | | | | | | |
| **Martin at al, 2013** | [Can transcranial direct current stimulation enhance outcomes from cognitive training? A randomized controlled trial in healthy participants.](https://www.ncbi.nlm.nih.gov/pubmed/23719048) | Adaptive dual *n*-back paradigm – participants had to respond to both auditory and visual streams of stimuli | 10 daily sessions | 54 participants were randomly assigned to a group that would receive either an active or sham tDCS along with a dual n-back task or tDCS alone. | tDCS | Participants in the active tDCS+CT condition performed more accurately on the CT task than participants who received the sham tDCS+CT. |
| **Martin et al, 2014** | [Use of transcranial direct current stimulation (tDCS) to enhance cognitive training: effect of timing of stimulation.](https://www.ncbi.nlm.nih.gov/pubmed/24992897) | Anodal tDCS to the left dorsolateral prefrontal cortex immediately before ('offline' tDCS) and during performance ('online' tDCS) on a dual n-back WM task, in an intra-individual crossover design. | 2 x 30-minute sessions over two days | 20 participants, 12 male, mean age 22.8 years (SD = 3.2) | tDCS | Results showed that 'online' tDCS was associated with better within session skill acquisition on the WM task, with a significant difference found between conditions the following day. These results suggest that 'online' tDCS is superior to 'offline' tDCS for enhancing skill acquisition when combining anodal tDCS with WM training. |
| **Jones et al., 2017** | Frontoparietal neurostimulation modulates working memory training benefits and oscillatory syncronization | Change detection WM task | 5 sessions in one week | 24 neurotypical students (mean age 24 years), active tDCS (5 females), sham tDCS (6 females) | tDCS and EEG | Those who had anodal tDCS experienced greater improvement on the WMT, compared to sham tDCS, and that this improvement was reflected in frontal-posterior alpha band power, and theta and low alpha oscillations associated with greater neural synchronisation. |
| **Stephens et al., 2016** | Older Adults Improve on Everyday Tasks after Working Memory Training and Neurostimulation | A subtract 2 span [[35](https://www.ncbi.nlm.nih.gov/pmc/articles/PMC4957521/#R35)], automated Operation Span (O-SPAN), and  spatial recall task, 5 items were presented and after a delay (4000 ms.), 12 images appeared. Participants clicked on the locations occupied during stimulus encoding. In the visual recall task, 5 items were presented on a computer monitor and after a delay (500 ms.), 16 items appeared. Participants selected the 5 items in the stimulus array. Both tasks had infinite time for participant response. | 5 sessions in one week | 90 healthy older adults randomly assigned to Sham (16 females, age 70 years), Active 1 (16 females, age 69 years), Active 2 (17 females, age 69 years) | tDCS, EEG and fNIRS | 2 mA of tDCS induced significantly greater far transfer gains one month after training. Moreover, these cognitive gains were observed on far transfer tasks (subtract 2 Letter Span Task, automated Operation Span and a spatial and visual WM task), and stimulation was well tolerated by all participants. Of note, for the first time the authors also utilised functional near infra-red spectroscopy (fNIRS) to examine potential neural changes associated with WMT, but were unable to find conclusive evidence. |
| **Stephens et al., 2017** | Task demands, tDCS intensity, and the COMT val158met polymorphism impact tDCS-linked working memory training gains | Visual and Spatial WM training paired with tDCS (sham, 1, 1.5, 2 mA). | 10 day and 5 day training study | 137 healthy older adults | tDCS and genotyping | Those with the val/val COMT genotype gained most from 1.5 mA tDCS during visual WMT, and from 1 mA tDCS during spatial WMT. For met/met polymorphisms, 2 mA resulted in significantly poorer performance compared to 1.5 mA on spatial WMT. The authors’ concluded that variations in COMT val158met may predict the nature of WM improvement after initial and longitudinal tDCS. |
| **Nissim et al 2019** | Effects of transcranial direct current stimulation paired with cognitive training on functional connectivity of the working memory network in older adults | N-back task (2-back versus 0-back) | 10 sessions of cognitive training paired with active or sham-tDCS | 28 healthy older adults (mean age 74) | tDCS | Active-tDCS vs. sham demonstrated a significant increase in connectivity between the left dorsolateral prefrontal cortex and right inferior parietal lobule at post-intervention during 2-Back. Target accuracy on 2-Back was significantly improved for active vs. sham at post-intervention. |
| **EEG/Cerebral profusion Studies (n=4)** | | | | | | |
| **Oelhafen et al, 2013** | [Increased parietal activity after training of interference control.](https://www.ncbi.nlm.nih.gov/pubmed/23982078) | High or low interference training variant of the dual n-back task, or a passive control group. | 14 x 25-minute home training sessions over three weeks (5 days of consecutive training and 2 days off) | 43 participants (mean age: 25.2 years). Both training groups consisted of 14 participants (6 female), and 15 people were assigned to the control group (8 female) | EEG | N-Back training with high interference led to some improvements in the Attention Network Test (ANT) but not to measures of working memory and fluid intelligence. The study also observed higher electrophysiological activity in the parietal cortex which the authors suggest may be demonstrative of the observed improvements in processing speed, attentional control, or both. |
| **Sun et al, 2014** | [Topological changes of the effective connectivity during the working memory training.](https://www.ncbi.nlm.nih.gov/pubmed/25571423) | spatial N-Back paradigm | 2 x 20-minute sessions over three days. | 2 healthy participants (age 32 & 60) | EEG | The neuroimaging revealed significant decreased clustering coefficient and normalized shortest path length, suggesting a reduced local efficiency with an increased global efficiency after WM training |
| **Jones et al. (2017)** | Frontoparietal neurostimulation modulates working memory training benefits and oscillatory synchronization | Change detection task completed during EEG/tDCS sessions. Participants indicated whether or not a target object had been seen previously among an array of common objects | 4 days | 24 healthy participants assigned to active (n=12, 5 females, 7 males) or sham (n=12, 6 females, 6 males) tDCS. | EEG/tDCS | Anodal tDCS demonstrated significant improvement on the WM task, compared to sham stimulation, mirrored by neural correlates in spectral and phase synchrony analyses of the HD-EEG data. The behavioral interaction corresponded to interactions in frontal-posterior alpha band power, and theta and low alpha oscillations. This suggests that that tDCS + WM training may enhance cortical efficiency and connectivity in task-relevant networks. |
| **Chen et al., 2019** | Distinguishing the visual working memory training and practice effects by the effective connectivity during n-back tasks: A DCM of ERP study | Memory Matrix N-Back task online | 5 x 30-minute sessions, 5 days per week for 3 weeks. | 20 right-handed healthy participants in 2 groups of 10, the WM training group (mean age 23.9, 2 females) and control group (mean age 24.5, 1 female) | EEG | Visual WM training alters the frontal-parietal connections, which comprise the executive control network (ECN) and the dorsal attention network (DAN), whereas practice modulates the parietal-frontal connections underpinning P300 production for selective attention |
